# Fungal infection drives metabolic reprogramming in epithelial cells via aerobic glycolysis and an alternative TCA cycle shunt

**DOI:** 10.1126/sciadv.aea0405

**Published:** 2026-02-04

**Authors:** Aize Pellon, Shervin Dokht Sadeghi Nasab, Gholamreza Bidkhori, James S. Griffiths, Stefania Vaga, Neelu Begum, Mariana Blagojevic, Nitesh Kumar Sigh, Natalia K. Kotowicz, Ifeanyi Uzochukwu, Adrien Le Guennec, Rhonda Henley-Smith, Harry Gregson-Williams, Frederick Clasen, Miranda Pryce, Nadia Karimpour, Richard Cook, Juan Anguita, Jonathan P. Richardson, Selvam Thavaraj, Julian R. Naglik, Saeed Shoaie, David L. Moyes

**Affiliations:** ^1^ Centre for Host-Microbiome Interactions, Faculty of Dentistry, Oral & Craniofacial Sciences, King’s College London, London, UK.; ^2^ Inflammation and Macrophage Plasticity Laboratory, CIC bioGUNE-BRTA (Basque Research and Technology Alliance), Derio, Spain.; ^3^ QIAGEN Aarhus, Aarhus, Denmark.; ^4^ NMR Facility, Guy’s Campus, King’s College London, London, UK.; ^5^ King’s Health Partners, Head and Neck Cancer Biobank, Guy’s and St. Thomas’ NHS Foundation Trust, London, UK.; ^6^ Department of Head and Neck Pathology, Guy’s and St. Thomas’ NHS Foundation Trust, London, UK.; ^7^ Cancer Metabolism Laboratory, The Francis Crick Institute, 1 Midland Road, London NW11AT, UK.; ^8^ Centre for Oral, Clinical and Translational Science, King’s College London, London, UK.; ^9^ Department of Oral Medicine, Floor 22 Tower Wing, Guy’s Hospital, Great Maze Pond, London SE1 9RT, UK.; ^10^ Guy’s and St Thomas’ NHS Foundation Trust, Great Maze Pond, London SE1 9RT, UK.; ^11^ Ikerbasque, Basque Foundation for Science, Bilbao, Spain.; ^12^ Quantitative Systems Biology, Faculty of Medicine, Biruni University, Istanbul 34015, Turkey.

## Abstract

*Candida albicans*–induced immunometabolic changes drive complex responses in immune cells. However, whether and how *C. albicans* causes remodeling of oral epithelial cell (OEC) metabolism is unclear. Here, we use in vitro experiments and patient biopsies to demonstrate that OECs undergo metabolic reprogramming when infected by *C. albicans* independently of candidalysin secretion, increasing glycolysis and decreasing tricarboxylic acid (TCA) cycle activity. Glycolysis and glucose transport inhibition show that these pathways support OEC cytokine release, highlighting the partial control of antifungal epithelial immunity by cellular metabolism. However, glucose supplementation disrupts OEC responses both in vitro and in vivo, suggesting that the fungus benefits from these metabolic shifts and that increased aerobic glycolysis in OECs is detrimental. Genome-scale metabolic modeling predicted a shutdown of the TCA cycle and a previously unidentified role for glutamic-oxaloacetic transaminase 1 (GOT1) in response to *C. albicans*, which was subsequently shown to be important for OEC survival during infection. This study reveals a fundamental role for hexose metabolism and identifies a GOT1-mediated TCA cycle shunt in regulating OEC survival and immune responses during mucosal fungal infections.

## Teaser

*Candida* hijacks epithelial metabolism via glycolysis and GOT1-dependent TCA shunt, influencing antifungal defense.

## INTRODUCTION

The control of immune responses by cellular metabolism, namely, immunometabolism, has emerged as a critical field in immunology, explaining how energy metabolism and metabolic pathways balance immune responses to infections (*1*, *2*). Upon activation by pathogen-associated molecular patterns (PAMPs), innate immune cells such as macrophages increase their glucose consumption and processing via the so-called Warburg effect (*3*). Specifically, this process induces increased glycolysis under normoxic conditions with lactate as the final by-product (i.e., aerobic glycolysis). This event is usually accompanied by the disruption of the tricarboxylic acid (TCA) cycle and associated reduction in mitochondrial oxidative phosphorylation (OxPhos) (*4*). In turn, this metabolic shift contributes to reprograming of the transcriptional landscape in activated cells through the accumulation of intermediate metabolites, such as succinate (*5*), or the direct effect of metabolic enzymes via their noncanonical (or moonlighting) functions, such as transcription/translation regulators (*6*–*8*). Notably, nonimmune cells such as epithelial cells may also undergo similar metabolic shifts in response to bacterial infections, but the broader implications of these shifts are unknown (*9*). Further studies of the metabolic regulation of immune responses in epithelial cells are critical to deciphering their relevance during common mucosal infections such as oral candidiasis, where oral epithelial cells (OECs) play pivotal roles in orchestrating antifungal immunity (*10*).

*Candida albicans* is a polymorphic fungal pathobiont considered a normal component of the human microbiome of multiple body sites (*11*–*13*). Under homeostatic conditions, *C. albicans* is a commensal organism. Although epithelial cells control commensal growth by regulating the fungal burden and recognizing morphological changes (*14*–*16*), alterations in environmental conditions and reduced host defenses promote invasive hyphal growth by *C. albicans*, resulting in localized infections and mucosal inflammation (*17*). As a consequence of immunosuppressive treatments or complex medical procedures, these superficial infections can potentially escalate to life-threatening systemic diseases. The high prevalence and morbidity of mucosal and systemic candidiasis make *C. albicans* the most clinically relevant fungal pathogen (*18*–*20*).

In recent years, our understanding of *C. albicans* pathogenesis in mucosal tissues has grown markedly because of the discovery of a secreted hyphal cytolytic toxin, candidalysin (*21*). Candidalysin is a critical driver of epithelial damage and immune responses (*21*, *22*). This toxin has also been linked to the activation of macrophages (*23*, *24*), endothelial cells (*25*), microglia (*26*), and platelets (*27*). The stimulation of epithelial cells with sublytic concentrations of candidalysin induces the release of proinflammatory factors, such as interleukin-6 (IL-6), granulocyte-macrophage colony-stimulating factor (GM-CSF), and IL-1β (*17*, *28*), as well as IL-36γ (*29*). In addition, candidalysin indirectly signals through the epidermal growth factor receptor and activates mitogen-activated protein kinase (MAPK) signaling (*30*–*32*), which subsequently promotes neutrophil recruitment and innate type 17 immunity (*33*).

To date, the role of epithelial metabolism during immune responses to *C. albicans* infection is poorly understood. As with other infectious agents or PAMPs, *C. albicans* reprograms monocyte/macrophage metabolism via C-type lectin receptors, promoting glucose consumption and aerobic glycolysis, thereby boosting anti-*Candida* immune responses (*34*, *35*). In turn, *C. albicans* competes for glucose with these cells, depleting this metabolite from the local environment and consequently inducing macrophage death through starvation (*35*). However, current knowledge is focused on the immunometabolic reprogramming of host immune cells during systemic infection. Hence, delineating the molecular mechanisms underlying immunometabolic changes in epithelial cells would substantially advance our understanding of mucosal fungal infections.

In this study, we combined molecular and cellular studies, with computational modeling and integrative omics analyses to delineate the immunometabolic changes in OECs in response to *C. albicans*. RNA sequencing (RNA-seq) and in vitro functional analyses of OECs infected with *C. albicans* showed a fungal-driven, candidalysin-independent metabolic shift toward aerobic glycolysis. This finding was confirmed in vivo in a murine model of oropharyngeal candidiasis (OPC) and biopsies from patients with oral candidiasis, with glucose uptake and processing being critical for developing functional immunity to the fungus. Subsequent simulations performed with genome-scale metabolic models (GEMs) led us to uncover a previously unknown, alternative pyruvate-processing pathway involving glutamic-oxaloacetic transaminase 1 (GOT1), which led to increased ammonia and aspartate levels in infected cells and a parallel dysfunction of the TCA cycle. These changes significantly affected anti–*C. albicans* epithelial responses, indicating that metabolic reprogramming plays a key role in host responses during mucosal candidiasis.

## RESULTS

### Candidalysin is the main driver of transcriptional alterations in OECs during *C. albicans* infection

**Fig. 1.**
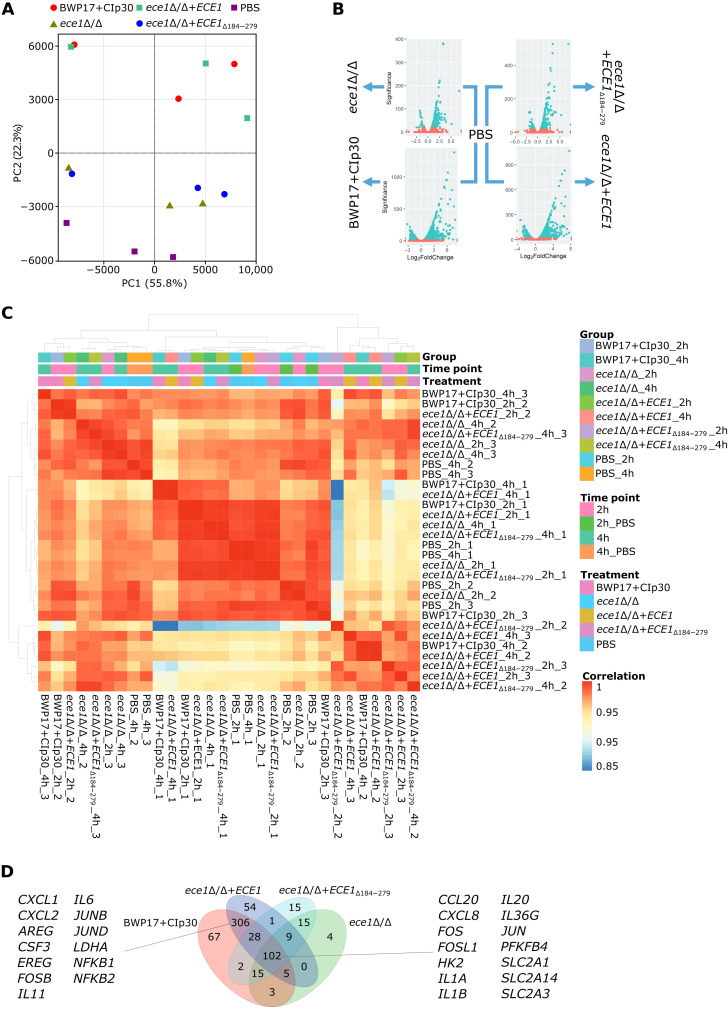
Transcriptomic profiles of OECs infected with *C. albicans*. (**A**) Principal components analysis (PCA) plot showing the relationship between samples based on their transcriptomic profiles at 4 hours postinfection. (**B**) Volcano plots depicting the number of differentially expressed genes (DEGs) in infected cells compared to cells exposed to PBS for 4 hours. (**C**) Heatmap showing the associations between the overall transcriptomic changes in analyzed samples. (**D**) Venn diagram depicting the number of DEGs in infected TR146 cells after 4 hours compared to PBS-stimulated cells.

To identify the pathways mediating host cellular responses to *C. albicans* and the impact of its peptide toxin candidalysin during mucosal infection, we explored the transcriptomic changes of the human OEC cell line TR146 infected with wild-type (BWP17+CIp30), candidalysin-null (*ece1*Δ/Δ and *ece1*Δ/Δ+*ECE1*_Δ184–279_), and revertant (*ece1*Δ/Δ+*ECE1*) mutant *C. albicans* strains for 2 or 4 hours. RNA-seq analyses globally showed greater transcriptional changes at 4 hours compared with 2 hours post–*C. albicans* infection (Fig. 1, A and B, and fig. S1A). Notably, the transcriptional profiles of OECs infected with fungal strains expressing candidalysin (BWP17+CIp30 and *ece1*Δ/Δ+*ECE1*) clustered together and separately from another cluster containing candidalysin-deficient strains (*ece1*Δ/Δ and *ece1*Δ/Δ+*ECE1*_Δ184–279_), both clusters being distinct from phosphate-buffered saline (PBS)–treated cells. Similarly, our analyses showed strong correlations in the transcriptome of OECs exposed to candidalysin-expressing strains at 4 hours postinfection, with fewer significant correlations in samples at 2 hours postinfection (Fig. 1C).

**Fig. 2.**
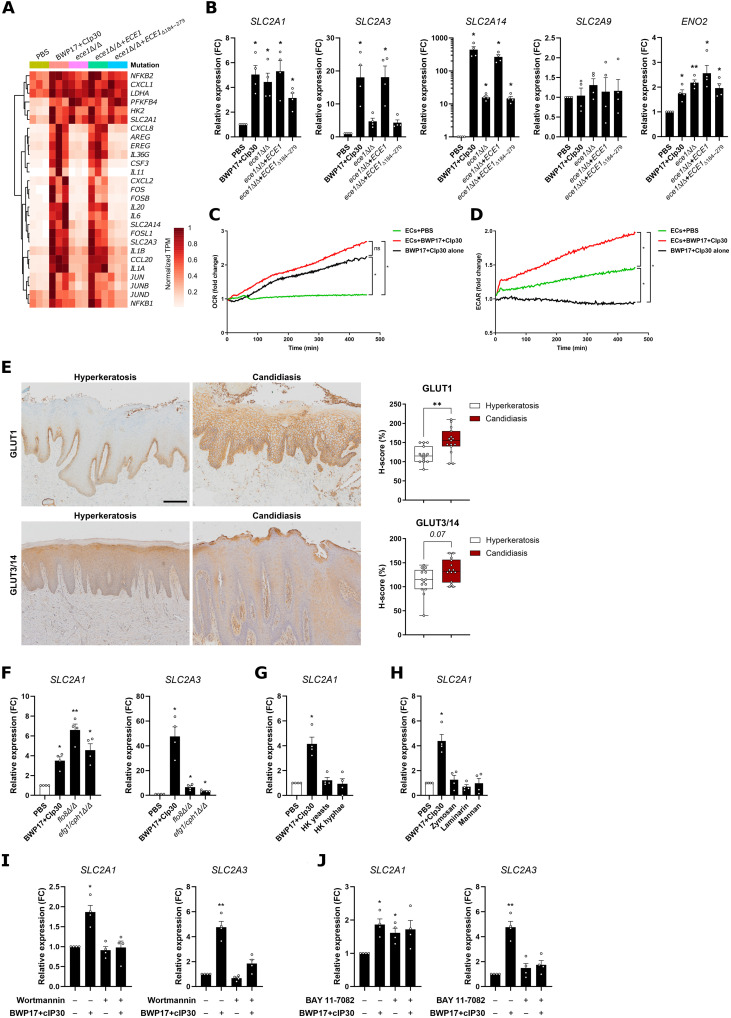
*C. albicans* infection induces a metabolic reprogramming toward aerobic glycolysis. (**A**) Heatmap depicting the changes in mRNA expression of selected genes related to immune response and metabolism in TR146 cells infected with the different *C. albicans* strains over noninfected epithelial cells. (**B**) RT-qPCR data of the indicated metabolic genes in *C. albicans*–infected TR146 cells for 4 hours [multiplicity of infection (MOI) = 10], relative to uninfected controls. Shown are the individual values and means ± SEM, *n* = 4. *P < 0.05; **P < 0.01; one-way analysis of variance (ANOVA). FC, fold change. (**C** and **D**) Oxygen consumption rates (OCRs) (C) and extracellular acidification rates (ECARs) (D) of *C. albicans* BWP17+CIp30 (MOI = 10), uninfected OECs, and BWP17+CIp30-infected OECs. Data are shown as the mean of the fold change of initial fluorescence values in three independent experiments. *P < 0.05; ns (not significant), *P* > 0.05; one-way ANOVA. (**E**) Immunohistochemistry photomicrographs and quantification of GLUT1 and GLUT14 expression in human oral epithelium biopsies from donors suffering from noninfection induced hyperkeratosis (*n* = 15) and oral candidiasis (*n* = 15). Scale bar, 200 μm. **P < 0.01; Mann-Whitney’s test. (**F** to **H**) RT-qPCR data of *SLC2A1* or *SLC2A3* expression in TR146 cells stimulated with BWP17+CIp30 (wild-type) strain, yeast-locked mutant strains *flo8*Δ/Δ or *efg1*/*cph1*Δ/Δ (F), heat-killed yeasts or hyphae (G), or conventional PAMPs, zymosan, laminarin, or mannan (H). Cells were stimulated for 4 hours with MOI = 10 for strains and 50 μg/ml for PAMPs. Shown are the individual values and means ± SEM, *n* = 4. *P < 0.05; **P < 0.01; one-way ANOVA. (**I** and **J**) RT-qPCR data of *SLC2A1* or *SLC2A3* expression in the presence of the PI3K (I) or NF-κB (J) inhibitor. Pathways were inhibited for 1 hour, and inhibitors were washed with warm PBS before *C. albicans* infection (MOI = 10) for 4 hours. Data are shown as individual values (*n* = 4) and means ± SEM. *P < 0.05; **P < 0.01; one-way ANOVA.

To uncover general, candidalysin-independent responses, we focused on a set of 102 genes that were significantly up-regulated [false discovery rate (FDR) < 0.01] in the presence of all four *C. albicans* strains 4 hours postinfection (Fig. 1D and table S1), suggesting that these might be key regulators of the early oral epithelium response to a fungal infection (0 to 4 hours postinfection). These included genes involved in the epithelial immune response to *C. albicans*, such as the proinflammatory cytokines *IL1A*, *IL1B*, and *IL36G*, the chemokines *CXCL8* and *CCL20*, and the transcription factors *JUN*, *FOS*, and *FOSL1*. Notably, although all 102 genes were differentially expressed in all groups compared to uninfected cells, some of them displayed a partial candidalysin-dependent response, as their expression was less prominent in strains with either partial (*ece1*Δ/Δ+*ECE1*_Δ184–279_) or complete (*ece1*Δ/Δ) *ECE1* deletion (Fig. 2A), in accordance with previous experimental observations (*21*, *28*, *29*). To gain a pathway-level information, the gene set enrichment analyses showed that 31.1% of all pathways that were significantly up-regulated (FDR < 0.001) under at least one condition were common among all the conditions (fig. S1B and data S1). Many of these up-regulated pathways are associated with cytokine signaling and inflammation. However, we found several Gene Ontology (GO) terms linked to metabolism up-regulated under all conditions, including “response to lipid,” “response to organic cyclic compound,” “response to ketone,” or “glucose transmembrane transport.”

### Central carbon metabolism in epithelial cells is regulated during *C. albicans* infection

Our transcriptomic profiling indicated that *C. albicans* induced metabolic reprogramming of epithelial cells. Most notably, hexose uptake and processing genes showed increased expression upon fungal infection (Fig. 2A). This group of genes included the glucose transporters *SLC2A1*, *SLC2A3*, and *SLC2A14*, along with the glycolytic enzymes *HK2*, *PFKFB4*, and *LDHA*. Furthermore, *SLC2A3* and *SLC2A14* showed partial regulation by candidalysin (Fig. 2A). We confirmed these transcriptomics data using reverse transcription quantitative polymerase chain reaction (RT-qPCR) for TR146 cells 4 hours postinfection with the same panel of fungal strains, confirming the up-regulation of all three glucose transporters (Fig. 2B). Further analyses using RT-qPCR showed no gene expression changes in the class II glucose transporter *SLC2A9* and the increased induction of the glycolytic enzyme gene *ENO2* by all strains (Fig. 2B). Similar trends for these genes were observed in the noncarcinoma DOK cell line (fig. S2A) and reconstituted oral epithelial models constructed with primary epithelial cells (fig. S2B). Notably, gene expression returned to basal levels 24 hours postinfection (fig. S2C). This metabolic shift was also observed when analyzing previously published time-course epithelial cell transcriptomics data from an OPC mouse model using both the *C. albicans* SC5314 (pathogenic strain) and 101 (commensal strain) (*36*). We observed increased gene expression of glucose transporters, glycolytic enzymes, and *Ldha*, which peaked 1 to 3 days postinfection and then declined (fig. S2D), suggesting that metabolic reprogramming of the epithelium might be transient.

Next, we addressed the functional consequences of gene up-regulation in epithelial cells upon infection. Challenge with *C. albicans* induced a significant (*P* < 0.05) and sustained increase in aerobic glycolysis in DOK cells (Fig. 2D), as measured by the extracellular acidification rate (ECAR), a methodology used as a proxy for glycolytic rate, as it measures the change in pH associated with increased lactic acid levels, the end product of aerobic glycolysis. This increased ECAR was induced by all four fungal strains (fig. S2E), suggesting that changes in ECAR are a candidalysin-independent event. Notably, the high oxygen consumption rates (OCRs) exhibited by *C. albicans* strains (Fig. 2D and fig. S2E) made it technically impossible to determine whether the fungus induced alterations in mitochondrial respiration or not.

To confirm that the *Candida-*induced metabolic reprogramming occurs in mucosal tissues during infection, we assessed the protein levels of key metabolic markers by immunohistochemistry in oral mucosal tissue biopsies from patients with chronic hyperplastic candidiasis (CHC) in comparison with noninfected hyperkeratosis tissue biopsies. In accordance with our in vitro observations, patients suffering from CHC showed increased membrane-associated expression levels of the glucose transporter GLUT1 (*P* < 0.001) (encoded by *SLC2A1*) in epithelial layers compared to individuals with hyperkeratosis. Similarly, the glucose transporter GLUT14 (*SLC2A14*) showed an increased expression trend (*P* < 0.0737) in patients with CHC (Fig. 2E).

Next, to shed light into the fungal components driving the metabolic shift, we performed experiments using yeast-locked mutant strains. Stimulation with either *flo8*Δ/Δ or *efg1*/*cph1*Δ/Δ up-regulated *SLC2A1* expression in OECs, while *SLC2A3* levels remained lower compared to the wild type, confirming the partial regulation of its expression by candidalysin (Fig. 2F). In contrast, neither heat-killed *C. albicans* (Fig. 2G) nor zymosan, laminarin, or mannan (Fig. 2H) induced *SLC2A1* overexpression. Last, we targeted two of the most relevant signaling molecules for anti–*C. albicans* epithelial responses (*10*) to assess their role in the regulation of the metabolic shift. Inhibition of phosphatidylinositol 3-kinase (PI3K) by wortmannin reduced both *SLC2A1* and *SLC2A3* expression (Fig. 2I), while nuclear factor κB (NF-κB) blockade only led to a decrease in Glut3 gene expression (Fig. 2J). These datasets reveal that *C. albicans* infection reprograms epithelial cell metabolism toward aerobic glycolysis via PI3K/NF-κB independently of morphological transition or candidalysin production, but relying on fungal viability.

### Hexose metabolism controls epithelial immune responses to *C. albicans*

**Fig. 3.**
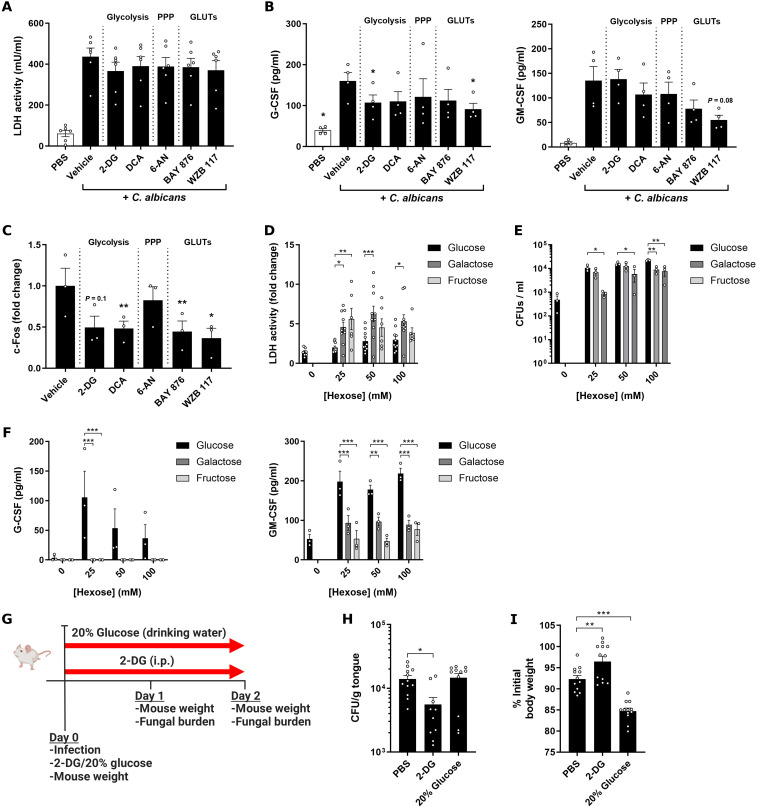
Hexose metabolism regulates OEC interactions with *C. albicans*. (**A** and **B**) TR146 cells were treated with metabolic pathway inhibitors for 1 hour, washed with warm PBS, and then infected with *C. albicans* BWP17+CIp30 (MOI = 0.01). Quantification of lactate dehydrogenase (LDH) activity (A), G-CSF, or GM-CSF (B) release 24 hours postinfection (p.i.). Data are shown as individual values (*n* = 4 to 6) and means ± SEM. *P < 0.05; one-way ANOVA. (**C**) TR146 cells were treated with metabolic pathway inhibitors and infected with *C. albicans* for 2 hours (MOI = 10). c-Fos expression was quantified by Western blot. Data are shown as fold change individual values (*n* = 3) and means ± SEM compared to vehicle-treated uninfected controls.*P < 0.05; **P < 0.01; one-way ANOVA. (**D** to **F**) TR146 cells were infected with *C. albicans* BWP17+CIp30 (MOI = 0.01) in the presence of varying concentrations of glucose, galactose, or fructose (0, 25, 50, and 100 mM). Quantification of LDH activity (D), fungal growth (E), and G-CSF or GM-CSF (F) production 24 hours postinfection. Data are shown as means ± SEM (*n* = 4 to 6). *P < 0.05; **P < 0.01; ***P < 0.001; two-way ANOVA. (**G**) Schematic of the murine OPC model experiment. ip, intraperitoneally. (**H**) Tongue fungal burden at day 2 postinfection in mice as shown in (G). Data are shown as individual values and means ± SEM (*n* = 11 to 12). *P < 0.05; one-way ANOVA. (**I**) Weight loss at day 2 postinfection in mice as shown in (G). Data are shown as individual values and means ± SEM (*n* = 11 to 12). **P < 0.01; ***P < 0.001; one-way ANOVA.

To determine the relevance of the *C. albicans*–induced metabolic reprogramming of OECs, we analyzed the impact of pharmacological inhibition of glucose transport, glycolysis, and pentose phosphate pathways on OEC responses. The inhibition of any of these pathways for 1 hour before infection had no significant impact on the *C. albicans*–induced damage [driven by candidalysin (*21*)] in either TR146 (Fig. 3A) or DOK (fig. S3A) cell line. The presence of the inhibitors during the course of infection did not have any impact on damage induction (fig. S3B) or fungal colony-forming units (CFU) (fig. S3C). However, the inhibition of glycolysis in the OECs alone using 2-deoxyglucose (2-DG) or of both glucose transporters (GLUT1 and GLUT3/14) using WZB 117 led to a significant decrease in granulocyte colony-stimulating factor (G-CSF) secretion (33 and 43% reduction, respectively; *P* < 0.05), with a nonsignificant reduction in GM-CSF production when all GLUT proteins were inhibited (Fig. 3B). The up-regulation of the activating protein 1 transcription factor component c-Fos (previously shown to be a key factor in the response to pathogenic *C. albicans*) (*14*) upon infection was hampered when either glycolysis (using 2-DG, *P* = 0.1, or dichloroacetate, *P* = 0.008), GLUT1 (BAY 876, *P* = 0.006), or GLUT1/3/14 (WZB 117, *P* = 0.04) activity was inhibited. However, the inhibition of the pentose phosphate pathway did not appear to affect c-Fos production (*P* = 0.75) (Fig. 3C). In contrast, no significant changes were observed in the phosphorylation patterns of the upstream MAPK extracellular signal–regulated kinase 1/2 (ERK1/2) or its regulatory phosphatase MAPK phosphatase (MKP1) (fig. S3D), which are also primarily driven by candidalysin (*21*). These results show that glucose transport and processing via glycolysis modulate the epithelial cell inflammatory output upon *C. albicans* infection via damage- and candidalysin-independent mechanisms.

The importance of glucose availability and homeostasis for innate immune cells during systemic candidiasis has been recently highlighted (*35*). Glucose deprivation, driven by the increased glucose consumption of *C. albicans*, leads to macrophage cell death in vitro and more deleterious disease outcomes in a systemic murine model. This phenotype is rescued in the presence of continuous, maintained glucose levels. In sharp contrast, we observed that *C. albicans* infection of TR146 cells led to an increasing trend in cell damage with higher glucose concentrations (Fig. 3D), which correlated with a higher fungal growth (Fig. 3E). Similar data were observed when lower, more physiological concentrations were used, including 5 mM (average concentration in serum) and 50 μM (average concentration in saliva), although the latter did not sustain OEC viability (fig. S3, E and F). Notably, when infections were performed with galactose or fructose as the sole hexose, even greater levels of epithelial damage were observed as compared to glucose (*P* = 0.006 and *P* < 0.001 for 25 mM galactose or fructose, respectively) (Fig. 3D). However, *C. albicans* growth in the presence of these hexoses was not as pronounced as in the presence of glucose. This shows an impaired damage protection response of OECs to *C. albicans* infection when galactose or fructose was the only hexose available. Accordingly, we observed a significant decrease in G-CSF and GM-CSF release in cells growing with galactose or fructose, with the complete abolition of G-CSF production in the presence of these hexoses (Fig. 3F). Notably, an absence of these carbon sources led to very low cell damage and cytokine release, despite the presence of *C. albicans* (Fig. 3, D to F). We ruled out a potential effect of osmolarity by incubating uninfected OECs in the presence of the different hexoses, which did not induce higher cell damage (fig. S3G) or cytokine release (fig. S3H). Furthermore, infections using the same concentrations of sorbitol only induced higher damage in noninfected cells at 100 mM, with no changes in CFU observed (fig. S3I). Overall, these data suggest that while glucose availability and utilization are essential for generating immune responses to *C. albicans*, elevated concentrations of sugars in the environment might lead to more deleterious disease outcomes for superficial *C. albicans* infections.

To further study the impact of hexose metabolism and availability, we performed in vivo experiments using a murine model of OPC. Mice were infected with *C. albicans* without any other treatment (“OPC” group), with intraperitoneal administration of 2-DG (“OPC + 2-DG” group), or with 20% glucose supplementation in the drinking water (“OPC + 20% glucose” group) (Fig. 3G). Notably, treatment with 2-DG delayed OPC establishment at day 1 postinfection (fig. S3K) and significantly decreased tongue fungal burden (*P* = 0.01) and weight loss (*P* = 0.009) compared to controls (Fig. 3, H and I). In contrast, mice supplemented with glucose showed a significant decrease in body weight (*P* < 0.001) despite no overall change in fungal burden (Fig. 3, H and I), with some mice being culled 2 days postinfection after reaching the humane end points (fig. S3J). RNA expression analyses of mice tongues showed a decrease in G-CSF (*Csf3*) expression in infected mice receiving 2-DG at day 2 postinfection (fig. S3L), despite no change was observed at day 1 (fig. S3M), suggesting a role of neutrophils. Neutropenic mice subjected to OPC (fig. S3N) did not show any alteration in fungal burden or body weight when given 2-DG compared to controls (fig. S3O).

### *C. albicans* alters the epithelial metabolome

Given the observed alterations in the expression of glucose transporter and glycolytic enzyme genes in epithelial cells due to *C. albicans* infection and the impact of metabolic pathway inhibition on the immune responses of epithelial cells, we next used genome-scale metabolic modeling in conjunction with our RNA-seq data to explore the impact of *C. albicans* infection on metabolic pathways (fig. S4A). We generated two context-dependent GEMs for *C. albicans*–infected and noninfected epithelial cells. The simulations identified key characteristics of the Warburg effect in infected OECs, with notable alterations in central carbon metabolism. The *C. albicans*–infected epithelial GEM showed an increased flux of sugar consumption (i.e., glucose, galactose, and fructose, among others), leading to increased pyruvate production. In contrast, the flux through the TCA cycle was substantially decreased. Our model also suggested that a key event resulting from increased pyruvate production was the elevated production of oxaloacetate caused by the actions of pyruvate carboxylase (PC). Notably, downstream analyses indicated a higher level of GOT1/GOT2 transaminase activity, with knock-on effects on glutamate/aspartate accumulation and ammonia metabolism. Amino acid metabolism was also predicted to change substantially, leading to higher glutamate synthesis in infected cells. These data suggest that the metabolic reprogramming of OECs induced by *C. albicans* infection involves not only increased aerobic glycolysis, as supported by our in vitro and in vivo experiments, but also decreased flux into the TCA cycle and alterations in glutamate metabolism.

**Fig. 4.**
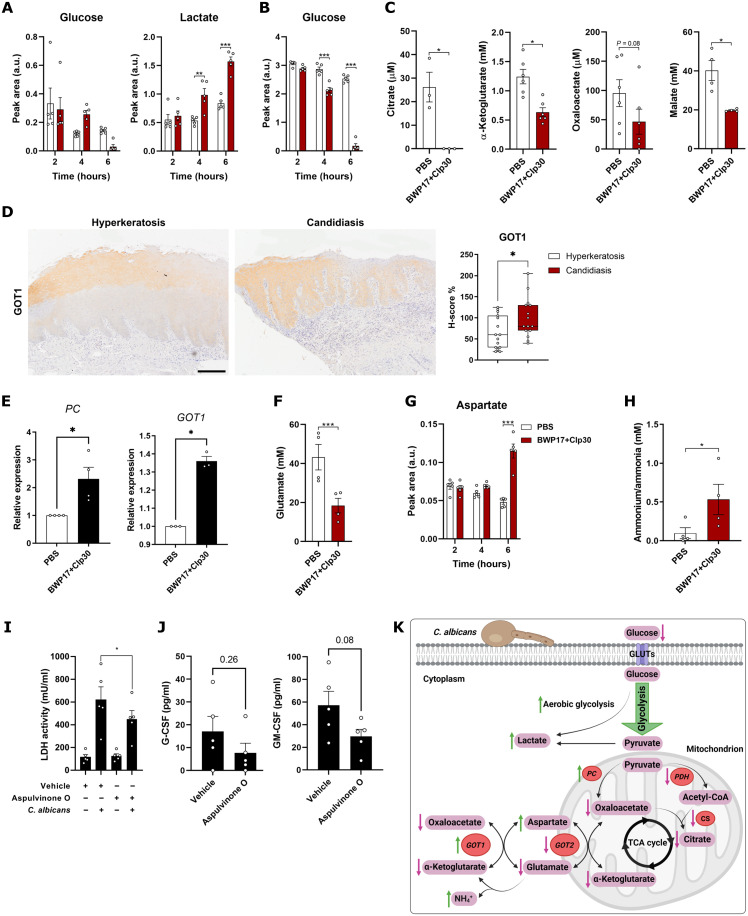
*C. albicans*–induced metabolome alterations in OECs. (**A** and **B**) Metabolomic analysis of OECs infected with *C. albicans* BWP17+CIp30 (MOI = 10) for 2, 4, or 6 hours (h) showing changes in intracellular glucose and lactate levels (A), and glucose in the exhausted culture medium (B). Data are shown as individual values and means ± SEM (*n* = 6). **P < 0.01; ***P < 0.001; two-way ANOVA. (**C**) Abundance of TCA cycle–associated metabolites decreased in *C. albicans*–infected TR146 cells (MOI = 10, 4 hours), including citrate, α-ketoglutarate, oxaloacetate, and malate (all detected using specific kits). Data are shown as individual values and means ± SEM (*n* = 4 to 6). *P < 0.05; Student’s *t* test. (**D**) Immunohistochemistry photomicrographs and quantification of GOT1 expression in human oral epithelium biopsies from donors suffering from noninfection induced hyperkeratosis (*n* = 15) and oral candidiasis (*n* = 15). Scale bar, 200 μm. *P < 0.05; Mann-Whitney’s test. (**E**) mRNA expression levels of *PC* and *GOT1* in TR146 cells infected with *C. albicans* (MOI = 10) for 4 hours. Data are shown as individual fold change values and means ± SEM (*n* = 3). *P < 0.05; Student’s *t* test. (**G** to **H**) Alterations in glutamate (F), aspartate (G), and ammonia (H) levels in infected cells (MOI = 10, 4 hours) OECs compared to uninfected controls. Data are shown as individual values and means ± SEM (*n* = 4 to 6). *P < 0.05; ***P < 0.001; two-way ANOVA (F) or Student’s *t* test (G and H). (**I** and **J**) TR146 cells were treated with the GOT1 inhibitor aspulvinone O for 1 hour, then washed with warm PBS, and infected with *C. albicans* BWP17+CIp30 (MOI = 0.01). LDH activity (I) or GM-CSF release (J) was quantified 24 hours postinfection. Data are shown as individual values and means ± SEM (*n* = 5). *P < 0.05; one-way ANOVA (I) or Student’s *t* test (J). (**K**) Schematic of the main metabolic alterations observed in OECs in response to *C. albicans* infection.

To test these in silico predictions, we used untargeted nuclear magnetic resonance (NMR) metabolomics to probe the epithelial metabolome during *C. albicans* infection. As predicted, we observed lower levels of glucose and higher levels of lactate in the intracellular compartment over time in *C. albicans*–infected epithelial cells (Fig. 4A), as well as decreasing concentrations of glucose in the spent medium supernatant (Fig. 4B). In addition, nicotinamide adenine dinucleotide (NAD) and nicotinamide adenine dinucleotide phosphate (NADP) levels were increased and reduced, respectively (fig. S4B), suggesting that the metabolic reprogramming induced by *C. albicans* also alters the energy balance in OECs. In keeping with this observation, the adenosine diphosphate (ADP)/adenosine triphosphate (ATP) ratio decreased in infected cells (*P* = 0.0219) (fig. S4C), demonstrating higher energy production.

Next, we looked for alterations in the TCA cycle and glutamate metabolism, given that our predictions indicated that the influx toward this cycle was reduced, leading to glutamate accumulation. The abundance of the key metabolites involved in the TCA cycle was decreased in *C. albicans*–infected cells, as shown by untargeted NMR for fumarate (fig. S4D) and specific assays for citrate, α-ketoglutarate, oxaloacetate, and malate (Fig. 4C). The decreased abundance of these metabolites was not uniform (e.g., citrate levels were substantially lower than oxaloacetate or α-ketoglutarate levels). Therefore, we hypothesized that this could be related to the increased flux through PC and GOT1, involved in a pyruvate-processing shunt that bypasses the TCA cycle by producing α-ketoglutarate and aspartate from oxaloacetate and glutamate, as predicted by the GEM simulations (fig. S4A). GOT1 protein levels were up-regulated in patients with CHC (Fig. 4D). Furthermore, RT-qPCR analyses showed significantly increased expression of both *PC* (2.2-fold increase, *P* = 0.03; Fig. 4E) in TR146 cells and *GOT1* in TR146 (1.33-fold increase, *P* = 0.005; Fig. 4E) and DOK (1.25-fold increase, *P* = 0.04; fig. S4E) cells when infected with *C. albicans*, while no significant changes were observed for the classical TCA enzymes *PDHX* and *CS* or mitochondrial *GOT2* in TR146 (fig. S4F). These findings, coupled with a lower abundance of glutamate (Fig. 4F) and higher levels of aspartate (Fig. 4G) and ammonia (Fig. 4H), supported our in silico predictions of a reduced TCA cycle activity and a shift in metabolic flux toward ammonia production. Last, to assess the relevance of this shunt in pyruvate processing during the infection, we inhibited GOT1 activity using aspulvinone O. Notably, lactate dehydrogenase (LDH) activity decreased (27.5% reduction, *P* = 0.02; Fig. 4I) upon infection in aspulvinone O–treated cells. Likewise, we observed a decreasing trend in both G-CSF (75.1% reduction, *P* = 0.26) and GM-CSF (48.1% reduction, *P* = 0.08) release (Fig. 4J). These data indicate that this pyruvate-processing shunt is an important mechanism in driving cellular damage and immune evasion during *C. albicans* infection (Fig. 4K).

Collectively, the data presented here demonstrate the relevance of metabolic reprogramming in epithelial cell responses to *C. albicans* infection. Furthermore, we demonstrate an alternative ending for aerobic glycolysis via GOT1, as well as the critical role of this GOT1-mediated TCA cycle shunt for OEC survival and anti-*Candida* immune responses.

## DISCUSSION

Increasingly, OECs are recognized as essential in orchestrating efficient immune responses (*10*, *17*, *37*). The secreted toxin candidalysin is a critical fungal factor that drives epithelial cell damage and subsequent immune activation in multiple infection models (*28*) and is thus a central mediator of the host–*C. albicans* interaction. In this study, we confirmed the central role of candidalysin during *C. albicans* epithelial infection but have additionally identified immunometabolic alterations in epithelial cells that determine the infection outcome independently of the toxin. These metabolic alterations comprise pathways related to hexose transport and utilization, and a GOT1-mediated TCA cycle shunt, which is also involved in epithelial damage and immune responses.

Metabolic shifts in innate immune cells are essential in mediating inflammatory processes and protective antimicrobial activities (*38*–*40*). The engagement of different receptors by PAMPs, whole microbial cells, or endogenous molecules such as cytokines modulates metabolism via diverse mechanisms. Carbon central metabolism [including glycolysis/gluconeogenesis, the TCA cycle, pentose phosphate pathway (PPP), and OxPhos] is a key player during metabolic reprogramming, with the shift toward aerobic glycolysis [the Warburg effect (*3*)] being the hallmark of this process. This shift has been observed in macrophages challenged with fungi, including *C. albicans* (*41*). The stimulation of monocytes with the fungal cell wall component β-glucan leads to increased metabolic activity, promoting both aerobic glycolysis and respiration (*42*). Similarly, heat-killed *C. albicans* yeast cells induce both these catabolic processes and glutaminolysis, while stimulation with the hyphal form only induces aerobic glycolysis (*34*). However, macrophages challenged with live *C. albicans* show increased glycolytic activity alongside the decreased expression of mitochondria-related genes (*35*). In addition to macrophages, glucose consumption mediated by Glut1 activity is essential for functional immunity and fungal clearance by neutrophils (*43*, *44*). Our data in OECs demonstrate a metabolic shift toward aerobic glycolysis occurring during the early stages of *C. albicans* infection, with no significant changes in expression of OxPhos-related genes, while epithelial OCR levels were obscured by the high respiration rates exhibited by fungal cells. However, we have previously shown that *C. albicans* infection induces mitochondrial depolarization and reduced activity in a candidalysin-dependent manner in OECs (*45*), suggesting a reduction in OxPhos. In line with this, vaginal epithelial cells challenged with four *Candida* species (*C. albicans*, *Candida glabrata*, *Candida tropicalis*, and *Candida parapsilosis*) show an early, common response to all species of increased expression of OxPhos-related genes but exhibit dysfunctional mitochondrial morphology and activity (*46*).

Notably, our finding that hexose transport and glycolysis control the epithelial immune response to *C. albicans* is opposite to that observed during systemic candidiasis and at the cellular level in macrophages (*35*), monocytes (*34*), or neutrophils (*43*, *44*). The inhibition of glycolysis leads to more deleterious disease outcomes during systemic *C. albicans* infections, with increased organ fungal burdens (*34*). However, increased glucose availability supports innate immune cell antifungal activities both in vitro and in vivo by boosting their infection-induced shift toward aerobic glycolysis and preventing glucose depletion from the environment by *C. albicans* (*35*). In sharp contrast, our data showed that inhibiting glycolysis improved survival and reduced tongue fungal burdens in experimental murine OPC, while increasing glucose availability impaired host protection in vivo instead of supporting immune responses, as previously reported in macrophages (*35*). Notably, glycolysis inhibition by 2-DG reduced G-CSF expression, suggesting a decrease in neutrophil activation/recruitment. Supporting this, neutropenic mice treated with 2-DG did not show any disease regulation, further suggesting that inhibition of neutrophils might be beneficial for oral candidiasis, as previously observed in vulvovaginal candidiasis (*47*). On the other hand, impaired host protection when high glucose was given was not due to greater fungal growth, as tongue fungal burdens remained unchanged. Similar findings were observed in infected OEC monolayers, where higher concentrations of glucose slightly increased damage and fungal growth instead of rescuing epithelial cells from infection. Conversely, substituting glucose for the alternative hexoses, galactose and fructose, induced even greater damage and abolished cytokine release, despite fungal growth being lower compared to equivalent glucose treatments. This suggests that other metabolic pathways might be affected in the presence of these two hexoses and that further analysis should be performed in the future to unveil the underlying mechanism. In addition, in the absence of any hexose source, fungal infection did not lead to significant changes in cell damage or cytokine production, suggesting that glucose competition between host and fungus plays a limited role during epithelial infections, unlike in macrophages (*35*). Overall, these data demonstrate that shifting toward aerobic glycolysis in the presence of high sugar concentrations in the oral cavity is detrimental during oral candidiasis. These data are of clinical relevance and might explain why patients suffering from diabetes mellitus are prone to develop oral candidiasis (*48*), since these individuals show higher concentrations of glucose in their saliva (*49*).

In addition to the alterations in epithelial hexose transport and utilization during *C. albicans* infection, in silico and in vitro metabolomic analyses showed the down-regulation of TCA flux, resulting from the increased activity of a pyruvate-processing shunt mediated by GOT1, leading to an accumulation of aspartate and ammonia in infected epithelial cells. Our current understanding of aerobic glycolysis is that pyruvate is fermented to lactate/lactic acid for subsequent removal. However, this GOT1-dependent pyruvate shunt represents a previously unknown mechanism for the disposal of pyruvate that provides alternative metabolites for both the host and the fungus. Inhibition of this shunt through blocking GOT1 activity partially rescued the damage induced by *C. albicans*, suggesting that the fungus takes advantage of aspartate/ammonia production by host cells.

In conclusion, our study provides the framework for understanding the immunometabolic control of antifungal responses in OECs. Our data highlight the relevance of hexose metabolism, showing that increased glycolytic activity induced by fungal infection is detrimental for the host during oral candidiasis. We also identified a GOT1-dependent pyruvate shunt for the disposal of pyruvate during *C. albicans* infection. Modulating these host responses and the metabolic environment are promising fields of research for the future development of new therapies, providing alternatives to antifungal drugs in managing long-term at-risk individuals.

## MATERIALS AND METHODS

### Mammalian cell culture and inhibitors

The TR146 buccal epithelial squamous cell carcinoma line (*50*) was obtained from the European Collection of Authenticated Cell Cultures and cultured in Dulbecco’s modified Eagle’s medium:nutrient mixture F-12 Ham (DMEM:F-12; Gibco) supplemented with 10% fetal bovine serum (FBS) and 1% penicillin-streptomycin. The dysplastic noncarcinoma cell line DOK (*51*) was a gift from M. Tavassoli (King’s College London, UK). DOK cells were cultured in DMEM supplemented with 10% FBS, 1% penicillin-streptomycin, and 1% hydrocortisone. Cells were routinely tested for mycoplasma contamination using a mycoplasma-specific primer. Before stimulation, confluent cells were serum starved overnight, and all experiments were performed in serum- and antibiotic-free medium.

EpiOral primary OEC models were purchased from MatTek, USA. After arrival, cell culture inserts containing tissues were placed in six-well plates containing 1 ml of the provided culture medium and incubated overnight. Next day, the exhausted medium was replaced with fresh medium, and inserts were infected with 50 μl of either PBS or PBS containing 2 × 10^6^ *C. albicans* cells. After 4 hours, tissues were separated from the inserts and placed in lysis buffer for RNA extraction.

For metabolic pathway inhibition experiments, OECs were treated with inhibitors for PI3K (wortmannin; 1 μM), NF-κB (BAY 11-7082; 100 μM), GLUT1 (BAY 876; 25 nM), GLUT1/3 (WZB 117; 10 μM), hexokinase (2-Deoxy-D-glucose, 2-DG; 10 mM), pyruvate dehydrogenase kinase (sodium dichloroacetate, DCA; 1 mM), 6-phosphogluconate dehydrogenase (6-aminonicotinamide, 6-AN; 100 μM) and GOT1 (aspulvinone O; 10 μM) or with the same volume of vehicle (dimethyl sulfoxide or water) for 1 hour. Following inhibition, cells were washed twice with warm PBS and infected with *C. albicans* in serum-free DMEM:F-12.

For experiments with varying hexose concentrations, DMEM lacking glucose (Gibco) was used for the fungal inoculation of epithelial cells after an overnight serum starvation in glucose-containing medium. Glucose, galactose, or fructose was added exogenously at the desired concentration of 25, 50, or 100 mM, and modified media were filter sterilized before use. No FBS or antibiotic supplementation was included in these experiments.

### *C. albicans* strains and fungal PAMPs

*C. albicans* SC5314 (*52*), BWP17+CIp30 (*53*), the Ece1p-deficient strains *ece1*Δ/Δ and *ece1*Δ/Δ*+ECE1*_∆184–279_, the revertant *ece1*∆/Δ+*ECE1* strain, and the yeast-locked mutant strains *flo8*∆/Δ and *efg1*/*cph1*∆/Δ [all mutant strains were generated elsewhere (*21*)] were used in this study. *C. albicans* strains were grown in YPD medium (1% yeast extract, 2% peptone, and 2% dextrose) at 30°C overnight in a shaking incubator to stationary phase. Cultures were washed in sterile PBS and adjusted to the required cell density. *C. albicans* cells were heat killed by incubating cell suspensions at 56°C for 1 hour. For stimulation experiments using PAMPs, zymosan, laminarin, and mannan (all from Sigma-Aldrich) were used at 50 μg/ml.

### RNA isolation and analyses by RNA-seq and RT-qPCR

For RNA-seq, TR146 cells were infected with *C. albicans* strains or PBS for 2 or 4 hours at a multiplicity of infection (MOI) of 10 (10 fungal cells to 1 epithelial cell). Total RNA from oral epithelial monolayers was isolated using the NucleoSpin II kit (Macherey-Nagel, Thermo Fisher Scientific, UK) following the manufacturer’s instructions. RNA quality control was performed with the Agilent RNA 600 Nano Kit using a Bioanalyzer. Libraries were prepared using the TruSeq RNA Sample Preparation kit V2 (Illumina) according to the manufacturer’s protocol.

Paired-end raw sequencing data (FASTQ files) were aligned to human reference (GRCh38) from ensemble release 92 using “Kallisto” software (*54*) to quantify transcripts per million (TPM) and count values of the transcripts. The gene-level count and TPM values were calculated from the transcript-level abundance and counts using R package “tximport” (*55*). Differential expression analyses were performed on the basis of gene-level counts (having Entrez ID and the median TPM > 1 across all samples) using R package “DESeq2” and Wald test *P* value (FDR < 0.01). R package “org.Hs.eg.db” was used for ID mapping, and hierarchical clustering with Ward.D2 and Euclidean distance were used to cluster the samples on the basis of the similarity matrix. In addition, principal components analysis (PCA) that visualized using R package “ggplot2” was performed on the basis of the log-transformed TPMs. Gene set enrichment analysis was performed using Piano (*56*), and GO terms were retrieved from the Molecular Signatures Database. Transcriptomics data are deposited at the European Nucleotide Archive (ENA) under the project no. PRJEB61179.

For RT-qPCR experiments, cells were infected with *C. albicans* strains for 4 or 24 hours, and RNA was extracted as described above and treated with TURBO DNase (Ambion, Warrington, UK) to remove genomic DNA. cDNA was synthesized from deoxyribonuclease-treated samples using Reverse Transcriptase Superscript IV (Life Technologies). cDNA samples were used for qPCR with EvaGreen mix (Solis Biodyne) using a Corbett Rotorgene 6000 (Corbett Research, Cambridge, UK). Primers used were taken from the PrimerBank database (http://pga.mgh.harvard.edu/primerbank/) and are listed in table S1.

RNA-seq data from Kirchner *et al.* (*36*) (publicly available at National Center for Biotechnology Information BioProject accession no. PRJNA491801) were used to identify whether any genes of interest were differentially expressed in *C. albicans*–infected murine oral epithelium. Differentially expressed genes (DEGs) were requested from the authors of the original publication, and DEGs for time points 9 hours, 1 day, 3 days, and 7 days compared to naïve cells are presented here. The RNA-seq analysis pipeline from the original publication in short, involved alignment of quality-trimmed reads to GRCm38.p4 using STAR and read counts per gene locus were calculated with htseq-count (*57*). Read count data were normalized using edgeR with the trimmed mean of *M* values method, and DEGs were identified using the limma package (*58*).

### Metabolic profiling

OCRs and ECARs were measured in DOK cells differentially stimulated either with PBS, the candidalysin-expressing strains (BWP17+CIp30 or *ece1*∆/Δ+*ECE1*), or the candidalysin-deficient strains (*ece1*Δ/Δ or *ece1*Δ/Δ*+ECE1*_∆184–279_). Epithelial cells (2.5 × 10^4^ cells per well) were seeded in clear-bottom, black 96-well plates and incubated overnight to promote cell adhesion.

For OCR experiments, the MitoXpress Xtra Oxygen Consumption Assay kit (Agilent) was used following the manufacturer’s instructions. Briefly, cells were washed twice with warm PBS and stimulated with 90 μl of serum- and antibiotic-free media containing fungal cells or PBS alone. Then, 10 μl of reconstituted MitoXpress Xtra reagent was added to all wells, and they were quickly sealed using prewarmed HS mineral oil. Plates were immediately transferred into a FlexStation 3 (Molecular Devices) and kinetically read over 4.5 hours [excitation (Ex.), 380 nm;emission (Em.), 650 nm].

For ECAR assessments, the pH-Xtra Glycolysis Assay kit (Agilent) was used following the manufacturer’s instructions. Briefly, cells were washed twice with warm PBS and once more with warm respiration buffer. Cells were stimulated with 90 μl of respiration buffer with or without fungal cells, and 10 μl of reconstituted pH-Xtra reagent was added to all wells. Plates were immediately transferred into a FlexStation 3 (Molecular Devices) and kinetically read over 4.5 hours (Ex., 380 nm;Em., 615 nm).

### Immunohistochemistry

The use of human biopsy tissue in this study was carried out under Health Research Authority ethical approval (Research Ethics Committee ID 18/LO/0321 and Integrated Research Application System project ID of 234402). Human biopsy material (formalin-fixed, paraffin-embedded) from hyperkeratosis or CHC was retrospectively identified from pathology databases at Guy’s and St. Thomas’ NHS Foundation Trust. Demographics of patients’ samples are shown in table S1. Tissues were sectioned (5 μm in thickness), mounted on Superfrost Plus adhesive glass slides (Thermo Fisher Scientific), and placed on a hot plate for 1 hour. Tissues were deparaffinized using standard procedures, and antigen retrieval was carried out with optimized buffer solution (sodium citrate buffer, pH 6) and boiled at 95°C for 15 min. Subsequently, slides were washed three times with 1× tris-buffered saline (TBS; Sigma-Aldrich), 5 min each. To block endogenous peroxidase activity and avoid nonspecific background reactions, sections were incubated in 3% hydrogen peroxide solution for 20 min followed by a washing step. Subsequently, slides were partially drained using tissue and ringed using a delimiting “PAP” pen (S2002, Dako). To block nonspecific epitopes on the tissue samples, sections were incubated with 1% bovine serum albumin in TBS and azide (pH 7.6) for 2 hours. Primary antibodies at the appropriate concentrations were diluted in blocking buffer and incubated with tissues overnight at 4°C. Next day, slides were washed and then incubated with the appropriate secondary antibody at room temperature for 1 hour. Slides were rinsed with TBS, and color was developed for 3 to 7 min using 3,3′-diaminobenzidine solution (Pierce). Counterstaining was performed by dipping slides for 2 min in Mayer hematoxylin and washing under a running tap until clear. Optimal intensity of nuclear staining was checked by light microscopy. Slides were dehydrated, mounted with DPX (dibutylphthalate polystyrene xylene) mountant (Merck), and left to set before examination.

All cases were independently scored by two observers (S.D.S.N. and S.T.). An ordinate value of 0 to 3 was assigned to the intensity of membranous staining of the tissues. The percentage of each intensity was allotted to the entire epithelium surface within the whole-mount tissue section, and a product of each intensity value and its percentage stained within the epithelium surface was determined. An “*H*-score” was then determined using the following formula: [(1 × % cell intensity 1) + (2 × % cell intensity 2) + (3 × % cell intensity 3)]. Following independent scoring of each case, the interobserver *H*-score differences were evaluated. Where a difference in interobserver value for each case was <20, an average of the two scores was taken to produce a final score. Where two scores were >20 in difference, scorers agreed a consensus value on a multiheaded microscope.

### Cell damage assay and cytokine release quantification

Following incubation, exhausted culture medium was collected and assayed for LDH activity using the CytoTox 96 Non-Radioactive Cytotoxicity Assay kit (Promega) according to the manufacturer’s instructions. Recombinant porcine LDH (Sigma-Aldrich) was used to generate a standard curve.

Cytokine levels in exhausted culture medium were quantified using the Human G-CSF and GM-CSF Quantikine ELISA kits (Bio-Techne). The data were analyzed using cytokine standard curves to determine analyte concentrations.

### Western blotting

Epithelial cells were lysed using radioimmunoprecipitation assay buffer containing protease (Millipore, UK) and phosphatase inhibitors (Sigma-Aldrich, UK), left on ice for 30 min, and centrifuged for 10 min in a refrigerated microfuge. Supernatants were assayed for total protein using the BCA (bicinchoninic acid) protein quantitation kit (Thermo Fisher Scientific, UK). Protein (15 μg) was separated on 12% SDS–polyacrylamide gel electrophoresis minigels before transfer to nitrocellulose membranes (GE Healthcare, Amersham, UK). After probing with primary and secondary antibodies, membranes were developed using the Clarity Western ECL Substrate (Bio-Rad, UK) and read using a ChemiDoc system (Bio-Rad). Densitometry analysis was performed using Image Lab (Bio-Rad). Antibodies for c-Fos, p-MKP1, p-ERK1/2, and β-tubulin were from Cell Signaling Technology (UK), and goat anti-rabbit horseradish peroxidase–conjugated antibodies were from Jackson Immunologicals Ltd. (Stratech Scientific, UK).

### Murine model of OPC

BALB/c female mice (22 to 25 g) were infected with *C. albicans* BWP17+CIp30 wild-type strain, as previously described (*59*). Briefly, on day 0, mice were sedated with an intraperitoneal injection of ketamine (110 mg/kg) and xylazine (8 mg/kg) and inoculated by placing a swab soaked in a *C. albicans* (10^7^ CFU/ml) suspension in sterile saline sublingually for 75 min. On the same day, mice were either left untreated or given 20% glucose in the drinking water or injected intraperitoneally with 2-DG (100 mg/kg). Investigators checked on mice twice daily, and changes in body weight were recorded daily. On day 1 or 2 postinfection, mice were euthanized, and tongues were excised for fungal load assessment. To render mice neutropenic, cortisone acetate (225 mg/kg) was subcutaneously injected on day −1 and 1 of the experiments.

Animal infections were performed in dedicated animal facilities at King’s College London under UK Home Office Project License P292BBCE6. No method of randomization was used to allocate mice to the different experimental groups, and no statistical method was used to determine sample size before the experiment. Animals in the same cage were part of the same experimental group.

### NMR untargeted metabolomics and metabolite assessment

For NMR metabolomics, 4 × 10^6^ TR146 cells were seeded on 90-mm cell culture dishes and infected after serum starvation with *C. albicans* BWP17+CIp30 cells using an MOI of 10 or PBS alone. After the incubation times, samples of the exhausted culture medium were collected and directly frozen. Then, epithelial monolayers were washed once with warm PBS, and intracellular metabolites were extracted using a methanol/chloroform/water protocol. Briefly, cells were scraped in the presence of 2.2 ml of chilled methanol and transferred to 15-ml tubes. One volume of chloroform was added, and samples were mixed on a rocker at 4°C for 10 min. Then, 2.2 ml of Milli-Q water was added, and samples were mixed and left on ice for 10 min to allow the formation of a stable bilayer. Tubes were centrifuged at 1550 rpm and 4°C for 45 min, and the aqueous layer was collected and stored at −80°C.

Before NMR analysis, aqueous cell extracts were dried out in a refrigerated SpeedVac. Metabolites were resuspended in 600 μl of D_2_O solution with 100 mM Na_2_HPO_4_, 4 mM NaN_3_, and 5 mM total soluble protein (TSP) and transferred to 5-mm tubes. For cell culture supernatant samples, 540 μl of sample was mixed with 60 μl of the same buffer as for the aqueous extracts and transferred to 5-mm tubes. Both types of samples were analyzed on the Bruker Avance NEO 600 MHz equipped with the TCI Cryoprobe Prodigy (Bruker). Spectra were acquired at 298 K and consisted for each sample of a ^1^H PURGE (pulse sequence for the removal of GYration-induced errors) spectrum, with 128 scans, an acquisition time of 2.62 s, and a relaxation time of 4 s. After acquisition, the spectra were then phase corrected and baseline corrected, and the chemical shifts were referenced to the TSP peak at 0.0 parts per million.

Once acquired, the two spectra datasets were transferred into MATLAB, and using a custom program written within MATLAB, the spectra were aligned by the PAFFT (pairwise alignment using a fast Fourier transform) method and then normalized by probabilistic quotient normalization. For PCA and PLS-DA (partial least squares-discriminant) analyses, the spectra were scaled with the Pareto algorithm. Quantification was done with the aligned and normalized datasets. Metabolite identification was performed using two-dimensional spectra (total correlation spectroscopy and heteronuclear single-quantum coherence), and confirmed with the Human Metabolome Database (HMDB) and Biological Magnetic Resonance Bank (BMRB) libraries. For single metabolite quantification of citrate, α-ketoglutarate, oxaloacetate, malate, glutamate, ADP/ATP ratio (all from Sigma-Aldrich), and ammonia (Abcam) specific kits were used following manufacturer’s instructions.

### Genome-scale metabolic model

To generate condition-specific GEMs, we used MADE (*60*) and the TIGER toolbox (*61*) to integrate expression data using log fold changes and FDR obtained from the DESeq2 analyses as input into the Human Metabolic Reaction (HMR) model (*62*). Flux balance analysis was used as a mathematical constraint–based modeling approach for analyzing the flow of metabolites through the metabolic network. This flux balance approach was formulated as a single mixed-integer linear programming problem through the CPLEX solver.

### Statistical analysis

Statistical analyses of in vitro, in vivo, and human data were performed using GraphPad Prism version 9 (GraphPad Software, CA, USA). Details on the specific tests applied and *P* values for each dataset are described in figure legends. At least three biological replicates were performed to measure each parameter under each experimental condition.
