# Supplementary material for: Fungal infection drives metabolic reprogramming in epithelial cells via aerobic glycolysis and an alternative TCA cycle shunt
Source: Sci Adv. 2026 Feb 4;12(6):eaea0405. doi: 10.1126/sciadv.aea0405 (PMC12871460; doi:10.1126/sciadv.aea0405)
Supplement: Supplementary file 1 — Figs. S1 to S4 Table S1 Legend for data S1 [file sciadv.aea0405_sm.pdf]

Science Advances
AAAS

Supplementary Materials for

Fungal infection drives metabolic reprogramming in epithelial cells via aerobic glycolysis and an alternative TCA cycle shunt

Aize Pellon *et al.*

Corresponding author: Aize Pellon, aize.pellon@kcl.ac.uk; Saeed Shoaie, saeed.shoaie@kcl.ac.uk; David L. Moyes, david.moyes@kcl.ac.uk

Sci. Adv. **12**, eaea0405 (2026)
DOI: 10.1126/sciadv.aea0405

The PDF file includes:

Figs. S1 to S4
Table S1
Legend for data S1

Other Supplementary Material for this manuscript includes the following:

Data S1

Figure S1.

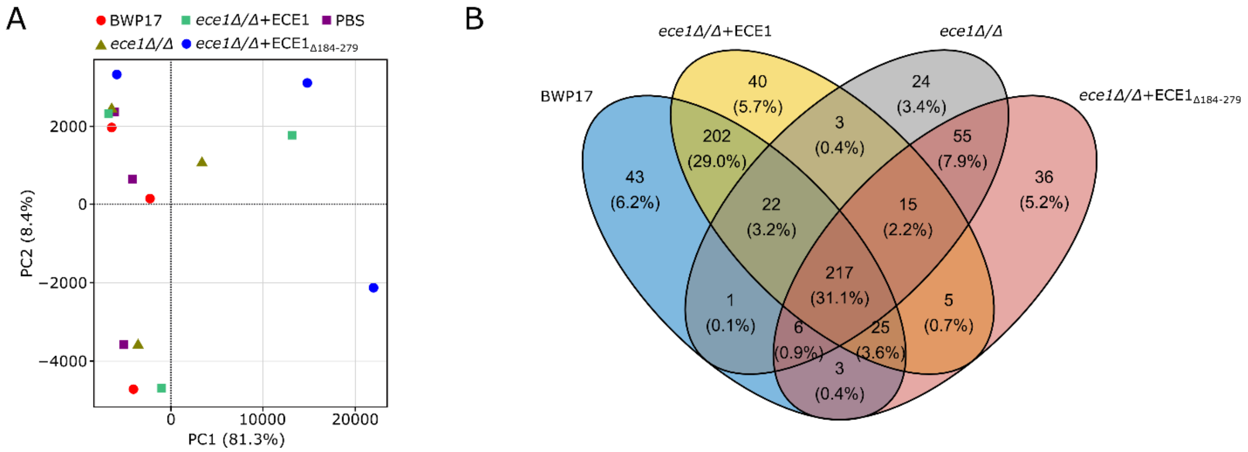

Fig. S1. *C. albicans* induces transcriptomic changes in oral epithelial cells.
(**A**) Principal Components Analysis (PCA) plot showing the relationship between samples based on their transcriptomic profiles at 2 h post-infection. (**B**) Venn diagram depicting the number of pathways observed by gene enrichment analyses in infected TR146 cells after 4 h compared to PBS-stimulated cells.

Figure S2.

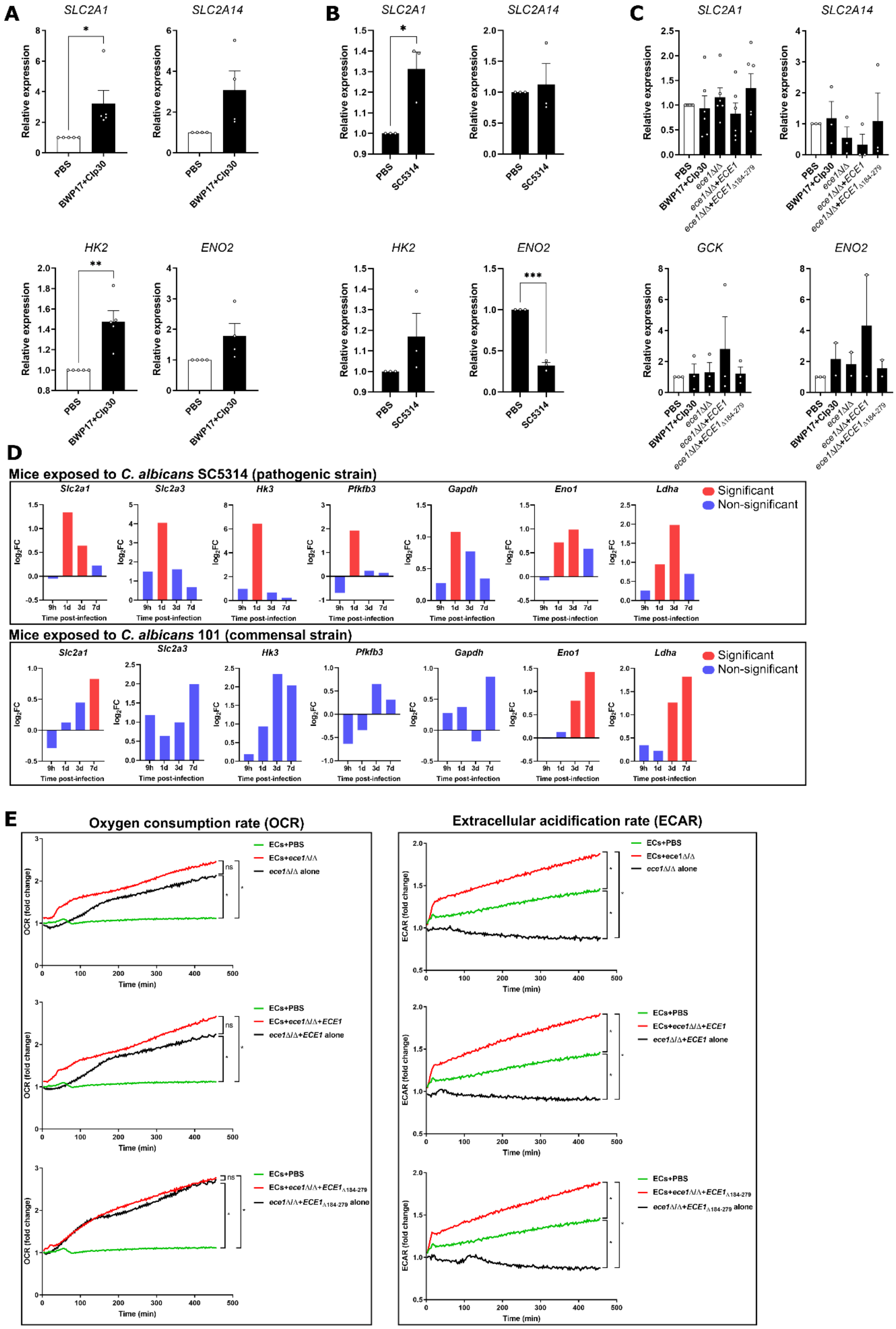
A
SLC2A1
SLC2A14
HK2
ENO2
Relative expression
PBS
BWP17+Clp30
B
SC5314
C
GCK
ece1Δ/Δ
ece1Δ/Δ+ECE1
ece1Δ/Δ+ECE1Δ184-279
D
Mice exposed to C. albicans SC5314 (pathogenic strain)
Slc2a1
Slc2a3
Hk3
Pfkfb3
Gapdh
Eno1
Ldha
Significant
Non-significant
log2FC
Time post-infection
Mice exposed to C. albicans 101 (commensal strain)
E
Oxygen consumption rate (OCR)
Extracellular acidification rate (ECAR)
OCR (fold change)
ECAR (fold change)
Time (min)
ECs+PBS
ECs+ece1Δ/Δ
ece1Δ/Δ alone
ECs+ece1Δ/Δ+ECE1
ece1Δ/Δ+ECE1 alone
ECs+ece1Δ/Δ+ECE1Δ184-279
ece1Δ/Δ+ECE1Δ184-279 alone
ns

Fig. S2. Glycolysis increases in response to *C. albicans* in the oral epithelium *in vitro* and *in vivo*.

(**A**) mRNA expression data of the indicated metabolic genes in DOK cells infected with *C. albicans* BWP17+Cip30 for 4 h, relative to uninfected controls. Data are shown as fold change individual values and mean ± S.E.M. compared to uninfected controls, and were analysed by Student's t-test, *$P<0.05$. (**B**) mRNA expression data of the indicated metabolic genes in primary oral epithelial cell models infected with *C. albicans* for 4 h, relative to uninfected controls. Data are shown as fold change individual values and mean ± S.E.M. compared to uninfected controls, and were analysed by Student's t-test, **$P<0.01$, ***$P<0.001$. (**C**) mRNA expression data of the indicated metabolic genes in TR146 cells infected with *C. albicans* for 24 h, relative to uninfected controls. Data are shown as fold change individual values and mean±S.E.M. compared to uninfected controls, and were analysed by one-way ANOVA, *$P<0.05$, **$P<0.01$, ***$P<0.001$. (**D**) Expression levels of selected metabolic genes in OPC experiments using *C. albicans* SC5314 (pathogenic strain) or 101 (commensal strain). RNA-Seq data were obtained from a previously published study *(36)* available at NCBI BioProject accession number PRJNA491801. (**E**) Changes in oxygen consumption (OCR) and extracellular acidification (ECAR) rates of oral epithelial cells in response to candidalysin-deficient strains (*ece1*Δ/Δ and *ece1*Δ/Δ+*ECE1*$_{\Delta 184\text{-}279}$) and the revertant *ece1*Δ/Δ+*ECE1* strain. Data shown as the mean of the fold change compared to initial fluorescence values in three independent experiments. *$P<0.05$; one-way ANOVA.

Figure S3.

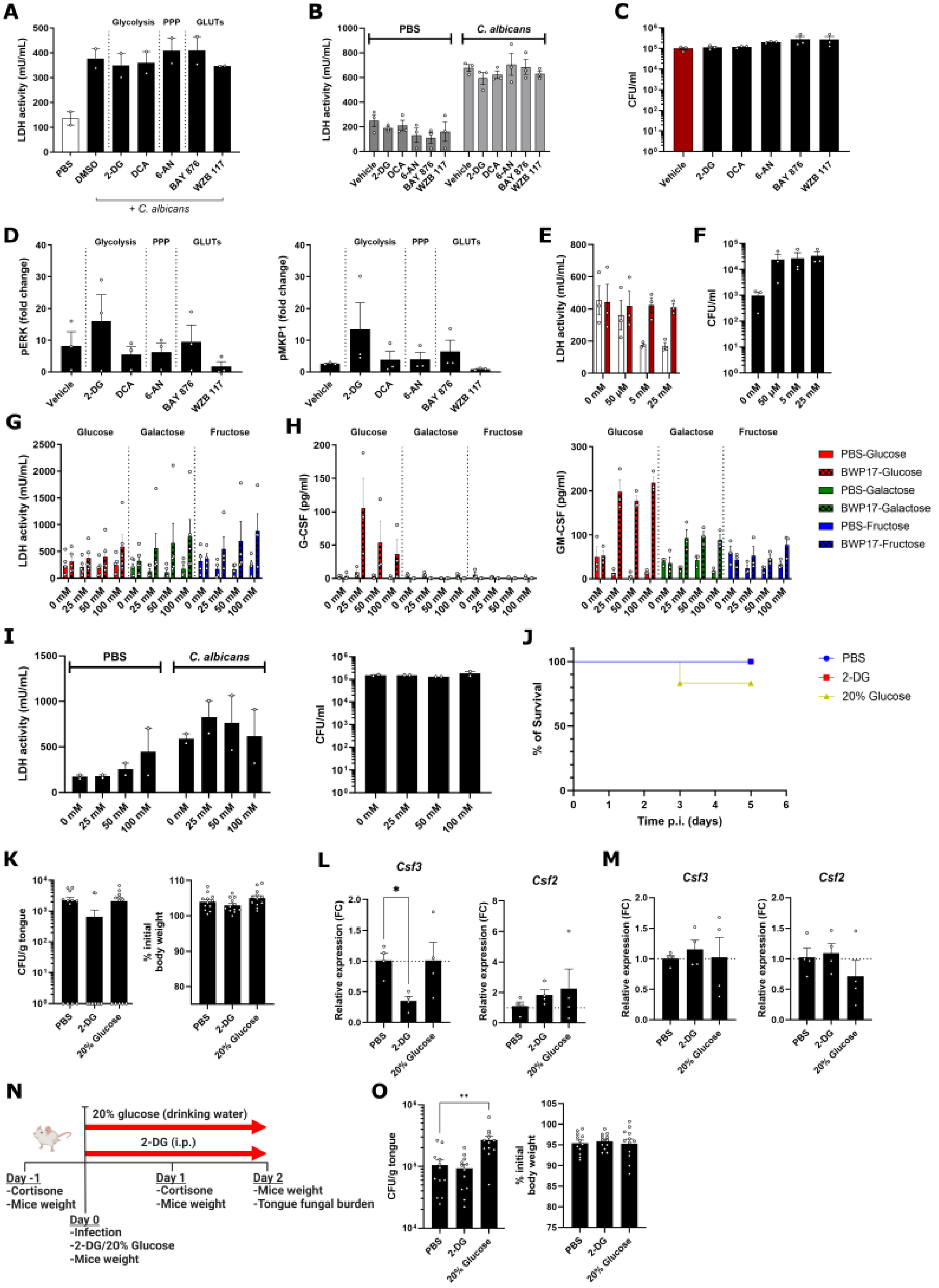
A
LDH activity (mU/mL)
Glycolysis
PPP
GLUTs
PBS
DMSO
2-DG
DCA
6-AN
BAY 876
WZB 117
+ C. albicans
B
PBS
C. albicans
Vehicle
C
CFU/ml
D
pERK (fold change)
pMKP1 (fold change)
E
F
0 mM
50 µM
5 mM
25 mM
G
Glucose
Galactose
Fructose
H
G-CSF (pg/ml)
GM-CSF (pg/ml)
PBS-Glucose
BWP17-Glucose
PBS-Galactose
BWP17-Galactose
PBS-Fructose
BWP17-Fructose
I
J
% of Survival
Time p.i. (days)
PBS
2-DG
20% Glucose
K
CFU/g tongue
% initial body weight
L
Csf3
Csf2
Relative expression (FC)
M
N
20% glucose (drinking water)
2-DG (i.p.)
Day -1
-Cortisone
-Mice weight
Day 0
-Infection
-2-DG/20% Glucose
-Mice weight
Day 1
-Cortisone
-Mice weight
Day 2
-Mice weight
-Tongue fungal burden
O

Fig. S3. Alterations in hexose metabolism and availability regulate anti-*C. albicans* responses in the oral epithelium.

(**A**) DOK cells were treated with metabolic pathway inhibitors for 1 h, then washed with warm PBS and infected with *C. albicans* BWP17+CIp30 (MOI=0.01). LDH activity was quantified 24 h post-infection (p.i.). Data are shown as mean ± S.E.M. and they were analysed by one-way ANOVA. (**B**, **C**) TR146 cells were treated with metabolic pathway inhibitors for 1 h before infection and then infected with *C. albicans* BWP17+CIp30 (MOI=0.01) without washing inhibitors away. LDH activity (**B**) and fungal growth (**C**) were quantified 24 h post-infection. Data are shown as individual values and mean ± S.E.M., and they were analysed by one-way ANOVA. (**D**) TR146 cells were treated with metabolic pathway inhibitors as in (**A**) and infected with *C. albicans* for 2 h (MOI=10). pERK1/2 and pMKP1 expression levels were quantified by western blot. Data are shown as fold change individual values and mean ± S.E.M. compared to vehicle-treated uninfected controls, and they were analysed by one-way ANOVA. (**E**, **F**) TR146 cells were stimulated with *C. albicans* BWP17+CIp30 (MOI=0.01) or PBS alone in the presence of varying concentrations of glucose (0, 50 μM, 5 mM, 25 mM). Quantification of LDH activity (**E**), and fungal growth (**F**) 24 h post-infection. Data are shown as individual values and mean ± S.E.M. (n=3). (**G**, **H**) TR146 cells were stimulated with *C. albicans* BWP17+CIp30 (MOI=0.01) or PBS alone in the presence of varying concentrations of glucose, galactose, or fructose (0, 25, 50, 100 mM). Quantification of LDH activity (**G**), and G-CSF or GM-CSF (**H**) production 24 h post-infection (p.i.). Data are shown as mean ± S.E.M. (**I**) TR146 cells were stimulated with *C. albicans* BWP17+CIp30 (MOI=0.01) or PBS alone in the presence of varying concentrations of sorbitol (0, 25, 50, 100 mM) and then LDH activity (left) and CFUs (right) were quantified. Data are shown as individual values and mean ± S.E.M. (n=2). (**J**) Survival curves during the oropharyngeal candidiasis (OPC) experiments using immunocompetent mice. (**K**) Tongue fungal burden and weight loss at day 1 post-infection in mice as shown in Figure 3G. Data are shown as individual values and mean ± S.E.M. (n=12). (**L**, **M**) mRNA expression levels of *Csf3* and *Csf2* in mice tongues of animals infected for 2 days (**L**) of 1 day (**M**). Data are shown as individual fold change values and mean ± S.E.M. (n=4). *$P<0.05$; one-way ANOVA. (**N**) Schematic of the immunocompromised murine oropharyngeal candidiasis (OPC) model experiment. (**O**) Tongue fungal burden and weight loss at day 2 post-infection in immunocompromised mice as shown in (**N**). Data are shown as individual values and mean ± S.E.M. (n=12). **$P<0.01$; one-way ANOVA.

Figure S4.

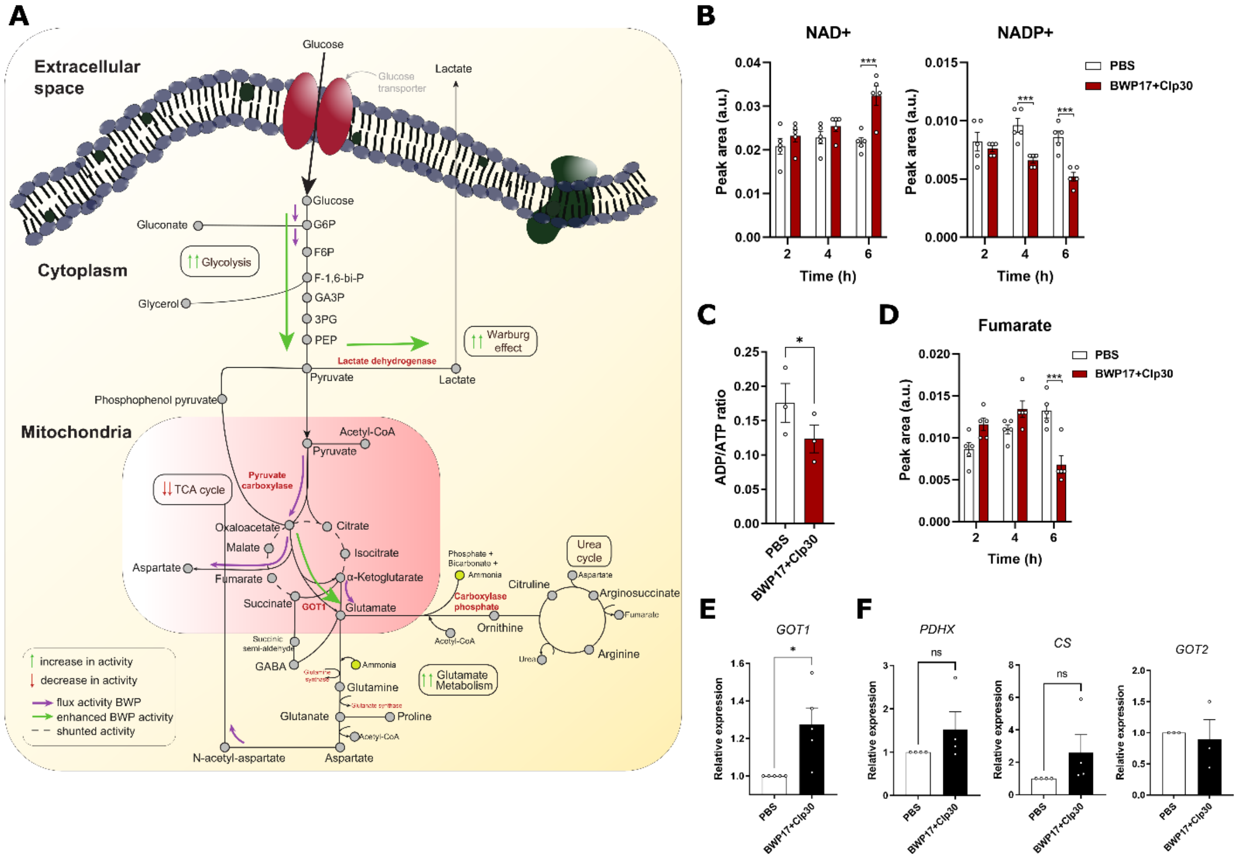

Fig. S4. *C. albicans* infection modifies glycolysis and TCA pathways in oral epithelial cells. (**A**) Schematic of the main metabolic pathways analysed in this study showing the predicted metabolic fluxes by GEMs. (**B-D**) Intracellular levels of NAD$^+$ and NADP$^+$ (**B**), ADP/ATP ratio (**C**), and fumarate (**D**). Data are shown as individual values and mean ± S.E.M. (n=3-6) and were analysed by two-way ANOVA, ***P*<0.01, ****P*<0.001; two-way ANOVA (**B**, **D**) or Student's t-test (**C**). (**E**) mRNA expression levels of *GOT1* in DOK cells infected with *C. albicans* (MOI=10) for 4 h. Data are shown as individual fold change values and mean ± S.E.M. (n=3). **P*<0.05; Student's t-test. (**F**) mRNA expression levels of *PDHX*, *CS*, and *GOT2* in TR146 cells infected with *C. albicans* (MOI=10) for 4 h. Data are shown as individual fold change values and mean ± S.E.M. (n=3).

Table S1. Demographic data from patients' samples. Metabolism-related markers were measured by immunohistochemistry (as shown in main figures 2 and 4) in oral epithelium biopsies obtained from the Head and Neck Cancer Biobank, Guy's & St Thomas' NHS Foundation Trust (London, United Kingdom). This tables shows the demographic data (age, sex and ethnicity) of the donors, as well as whether they suffered from oral candidiasis or non-infection-induced keratosis.

**Diagnosis**	**Year**	**Age at treatment**	**Sex**	**Ethnicity**
Candidiasis	2017	64	Male	Other or declined to answer
Candidiasis	2018	74	Female	White - Irish
Candidiasis	2018	62	Female	White - British
Candidiasis	2018	62	Female	White - British
Candidiasis	2018	63	Female	African/Afro-Caribbean
Candidiasis	2018	56	Male	African/Afro-Caribbean
Candidiasis	2018	45	Female	White - Any other
Candidiasis	2018	51	Male	Other or declined to answer
Candidiasis	2018	58	Male	Other or declined to answer
Candidiasis	2018	52	Female	White - English
Candidiasis	2018	57	Female	White - British
Candidiasis	2018	59	Male	Other or declined to answer
Candidiasis	2018	73	Female	Other or declined to answer
Candidiasis	2018	62	Male	Asian Indian/British Indian
Keratosis	2017	38	Male	White - British
Keratosis	2017	59	Female	Other or declined to answer
Keratosis	2017	61	Male	Asian
Keratosis	2017	57	Female	European
Keratosis	2017	46	Male	Afro-Caribbean
Keratosis	2017	48	Male	Black - Other African
Keratosis	2017	66	Female	Other or declined to answer
Keratosis	2017	37	Male	Asian
Keratosis	2017	62	Male	Afro-Caribbean
Keratosis	2017	59	Male	White - British
Keratosis	2017	55	Female	Other or declined to answer
Keratosis	2017	68	Female	Asian
Keratosis	2017	73	Male	White - English
Keratosis	2017	58	Male	Asian
Keratosis	2018	74	Male	Other or declined to answer

Supplementary Data S1. Pathway analysis of epithelial transcriptomic profiles in response to *C. albicans*. Pathway enrichment analysis of mock (PBS)-infected compared to *C. albicans*-infected TR146 cells. Each sheet shows data for each strain used in the transcriptomics analysis.
